# Internet Use on Closing Intention–Behavior Gap in Green Consumption—A Mediation and Moderation Theoretical Model

**DOI:** 10.3390/ijerph20010365

**Published:** 2022-12-26

**Authors:** Xintian Wang, Zhangchi Wang, Yan Li

**Affiliations:** School of Environment and Natural Resources, Renmin University of China, Beijing 100872, China

**Keywords:** green consumption, intention–behavior gap, Internet media, perceived environmental threats

## Abstract

The rapid development of the Internet as an information medium has provided new opportunities for promoting green consumption. Therefore, a study on the theoretical mechanism is helpful to make better use of the Internet media to promote green consumption and close consumers’ green consumption intention–behavior gap. In this study, data from 419 valid questionnaires were collected and analyzed through PLS-SEM within the framework of the theory of planned behavior. The results show that there are two pathways of Internet media promoting green consumption, namely the moderating effect and the mediating effect. First, through the moderating effect, Internet use can promote the conversion of intention to behavior and perceived behavioral control to behavior, thus closing the intention–behavior gap. Second, through the mediating effect, Internet use promotes green consumption behavior through the mediator of personal perceived environmental threats. The research indicates that the potential of Internet information media should be fully explored in promoting green consumption, disseminating environmental knowledge, reporting environmental issues, and guiding the transformation of individual green consumption intention into behavior.

## 1. Introduction

With the rising severity of environmental problems and scarcity of resources, previous extensive and wasteful consumption patterns have been questioned and challenged [1]. As a consumption pattern with positive effects on resources, the environment, and society, green consumption is gradually being recognized and accepted [2]. Individual consumption behaviors have large impacts on overall resource use and carbon emissions [3]. According to the IPCC 2020 Emission Gap Report, two-thirds of global carbon emissions are related to household consumption activities [4]. Thus, it is very important to promote individual green consumption behavior to reduce resource waste and promote carbon emission reduction. Results of interviews, surveys, and polls show that the majority of consumers have a positive attitude and intention toward green consumption [5,6,7]. However, previous studies also revealed the issue of the intention–behavior gap in green consumption, that consumers express a high willingness to purchase in a green manner, but few real actions have been taken [8,9]. The positive intention is difficult to transform into real behaviors [10]; thus, the existence of this inconsistency between intention and behavior hinders the transformation of an individual’s consumption pattern and has a negative impact on promoting sustainable development.

Previous studies have investigated how to close the green intention–behavior gap from the perspective of an individual’s psychological factors [11,12]. Zheng et al. [13] found that perceived environmental responsibility can impact green purchasing behaviors through the mediation of attitude. Ran and Zhang [14] found that price sensitivity can moderate the relationship between an individual’s recycling intention and recycling behavior. Some studies also found that besides internal factors, external and contextual conditions also have impacts on the gap [15,16]. Sultan et al. [17] stated that communication with consumers could moderate the relationship between their intention and behavior in terms of organic food purchasing. According to Nguyen et al. [10], green product availability also moderates the relationship between intention and behavior, therefore closing the gap. In particular, previous studies suggested that lack of information and relevant knowledge is one of the key barriers for consumers to implement actual green purchasing [9,18,19,20]. When consumers are not aware of or concerned about the environmental consequences [21,22] of their behaviors, they are less likely to perform green consumption behaviors. When perceived behavioral control increases, that is, consumers’ perception of their control over behavior consequences, the possibility of them conducting real behaviors also grows. Perceived behavioral control is also considered the single best predictor of behavior [23]. Therefore, more explicit and clear information about the consequences of green buying behavior can help bridge the intention–behavior gap [24,25,26]. Whether consumers have trust in the information is also related to the information’s effectiveness [27] as well as information simplicity and acceptability [28,29].

Internet media provides individuals with new access to environmental information [30]. With the growing penetration of Internet technology, more residents can acquire both positive and negative information related to environmental protection through Internet media [31]. Chi [32] found that social media has significant impacts on green consumption intention with the moderation of environmental concerns. Some scholars also found that Internet media can increase general pro-environmental behaviors, such as refuse classification, discussion of environmental issues with friends, donation for environment protection, etc., by encouraging participation in environmental protection actions [33], providing environmental knowledge [31,34] and improving environmental awareness [32,33].

Though some previous studies have noticed the important role of the Internet as an information medium in promoting general pro-environmental behaviors, few studies have investigated its role in green consumption behavior in the context of China (as shown in Table 1). Given the importance of individual green consumption behaviors and the differences in individuals’ decision-making processes on differentiated pro-environmental behaviors [35,36], it is of great significance to study the impact of Internet use as information media on specific green consumption behaviors. Furthermore, many studies investigated how to close the gap from the perspective of an individual’s intrinsic factors, such as price sensitivity [14], self-efficacy [37], and green values [38]. Contextual factors, especially Internet media use, await further investigation on their significance and mechanisms to close the gap. The underlying paths and theoretical explanation of the mechanism of how Internet media can contribute to closing the intention–behavior gap remains to be explored.

To address the above-mentioned gaps in the current literature, this study aims to develop a research framework based on the theory of planned behavior, discussing the mechanism of Internet media use impacting individual green consumption behavior and how it can act in closing the intention–behavior gap of green consumption. In terms of mechanism, this study integrates the variable of Internet use and perceived environmental threats into the framework to investigate the mediation and moderation effects.

This study complements previous research in the following three ways. First, this research focuses on green consumption instead of general pro-environmental behavior to explore the impact of the Internet as information media. Second, this research studied within the framework of the theory of planned behavior [42] to explore the decision-making mechanism, explaining how contextual factors, as well as psychological factors, impact consumers’ green consumption behavior. Third, the results of the study indicate that Internet use is a practical way to solve the issue of the consumer green consumption intention–behavior gap, providing implications that proper utilization of Internet media can promote the sustainable development of the consumption domain.

The remainder of the study is structured as follows. Section 2 discusses the TPB theoretical foundation of the study and proposes the hypotheses. Section 3 discusses the data collection, questionnaire development, survey conduction, and data analysis procedure. Section 4 provides data analysis results in terms of model reliability and validity as well as the results of the hypotheses. Section 5 discusses the results, provides implications accordingly, and addresses the study’s limitations. Section 6 concludes the study.

## 2. Theoretical Framework and Hypotheses

This study chooses Internet use, perceived environmental threats, intention, and perceived behavioral control to construct a theoretical framework within the paradigm of the theory of planned behavior. Internet use is an important external information factor impacting an individual’s behavior [31,33,34]. The theory of planned behavior (TPB), proposed by Ajzen in 1991 [42], is a theoretical model to explain an individual’s behavioral decision-making process. This model has been applied in many studies in the field of green consumption and has shown a good ability to explain consumers’ green consumption intention and green consumption behavior [17,43,44]. Attitude and subjective norms in the original TPB framework cannot directly impact behavior through intention. This study focus on the relationship between intention and behavior and how to convert intention into behavior. Therefore, referring to previous studies [14,45], core constructs are kept and adopted, namely intention and perceived behavioral control as predictors of behavior in this study. Perceived environmental threats are a psychological factor whose impacts on an individual’s pro-environmental behavior have been explored and tested in a few studies [31,46]. This study aims to construct a theoretical framework to study the mechanism of how Internet use can contribute to promoting consumers’ green purchasing behavior and closing the intention–behavior gap.

### 2.1. Theory of Planned Behavior and Intention–Behavior Gap

TPB follows the rational paradigm in studying consumer behavior; that is, an individual’s behavior follows the relationship of attitude–intention–behavior, and behavior is directly determined by intention [8]. However, intention only catches individual motivation to perform a certain behavior [42]; whether people can perform a certain behavior is also constrained by their ability to obtain relevant resources and opportunities (such as time, money, etc.) [47]. When consumers feel that they lack control over the consequences of performing a certain behavior and lack the corresponding resources and abilities, it is difficult for them to perform the corresponding behavior even if they have positive motivation [48], resulting in the intention–behavior gap. Therefore, the TPB model added the influencing factor of perceived behavior control (PBC) to the original rational model [42,47]. On the one hand, PBC measures the difficulties and obstacles that individuals perceive to prevent them from carrying out behaviors [49]. On the other hand, it also contains a concept similar to personal efficacy [47], that is, the ability of an individual perceives to manipulate the consequences of a certain behavior.

In summary, intention measures the strength of consumers’ motivation for an activity, while PBC reflects the difficulty consumers perceive in carrying out an activity and their control over the consequences of the behavior [50]. Though intention can hardly fully transfer into behavior, it is still an important antecedent of behavior [48,51]. PBC can affect behavior through two pathways, influencing behavior by enhancing intention and directly affecting behavior when people’s intention cannot completely control their behavior [52].

Therefore, the following hypotheses are proposed:

**Hypothesis** **1 (H1).** *PBC positively impacts green consumption intention.*

**Hypothesis** **2 (H2).** *PBC positively impacts green consumption behavior.*

**Hypothesis** **3 (H3).** *Intention positively impacts green consumption behavior.*

The TPB model includes both internal rational factors and ability factors of consumers’ decision-making process, but some scholars argue that the model neglects the influences of external conditions [1,53]. Previous studies suggest that external barriers, such as lack of information, distrust, and high prices are the main reasons for the gap between green intention and green consumption behavior [5,18,20]. Results of previous studies show that external facilitators can help close the intention–behavior gap [39,54]. Therefore, the next section will explore the role of Internet use as an external information factor in promoting green consumption.

### 2.2. Internet Use

Internet use greatly changes people’s psychological conditions [55] and behaviors [56], especially how people obtain knowledge and information during the COVID-19 pandemic [57]. Compared with traditional media, the Internet can provide information to individuals faster and more efficiently [58,59]. Information media can function to spread information and educate consumers [60]. Some studies show that media use can improve individuals’ awareness of environmental issues [37,40]. Due to the complex characteristics of the network, information dissemination on the Internet contains not only positive environmental protection news but also potentially negative news on threats from environmental degradation and pollution [61]. With increasing Internet use, Internet media have reported environmental pollution events more frequently and intensively than traditional media [33]. Lim and Moon [46] found that negative information on environmental pollution from the rising environmental problems will increase an individual’s PET. Liu et al. [31] further demonstrated that higher frequency Internet use can lead to higher perceived environmental threats. Therefore, more frequent use of the Internet increases the likelihood of consumers receiving negative information on environmental pollution and degradation, which in turn enhances their PET. Therefore, a hypothesis is proposed:

**Hypothesis** **4 (H4).** *Internet use positively impacts PET.*

From the theoretical analysis of the intention–behavior gap under the TPB framework, there are two possible pathways of how Internet use can promote individuals’ pro-environmental behaviors, including intention and PBC. Previous studies found that external factors can contribute to closing the intention–behavior gap through moderating effects, namely moderating intention–behavior and moderating PBC–behavior [17,19]. Grimmer and Miles [39] confirmed that favorable contextual factors could positively moderate the relationship between consumer intentions and pro-environmental behaviors. Sultan et al. [17] found that when consumers acquire information through communication, this external factor can positively moderate the relationship between intention and behavior as well as the relationship between PBC and behavior.

In terms of intention, consumers can have access to more environmental information through Internet use, which can enhance environmental awareness and willingness to participate in pro-environmental behaviors [33,62]. Chi [32] found that social media plays a significant role in promoting an individual’s green intention, and environmental concern modifies the relationship between social media use and intention. Anastasiei et al. [63] found that social media use can also influence consumption intention through electronic word-of-mouth. Environmental awareness is also considered an important influencing factor of consumers’ green consumption intention [22,64]. Thus, the following hypothesis is proposed:

**Hypothesis** **5 (H5).** *Internet use can positively moderate the relationship between intention and green consumption behavior.*

In terms of ability, previous studies confirmed that Internet use could promote an individual’s environmental knowledge [31,65]. Through mastering the necessary environmental knowledge for implementing pro-environmental behaviors and learning the approaches to participate in relevant activities through Internet use, an individual’s ability to perform actual behaviors is enhanced [65]. Karimi et al. [66] found that media use can enhance an individual’s PBC on green behaviors. Huang [37] found that more frequent exposure to global warming issues through media use can help transform one’s environmental self-efficacy into actual pro-environmental behaviors. Thus, the following hypothesis is proposed:

**Hypothesis** **6 (H6).** *Internet use can positively moderate the relationship between PBC and green consumption behavior.*

### 2.3. Perceived Environmental Threats

Perceived environmental threats (PET) refers to an individual’s assessment of the threats of environmental degradation [67]. Many studies confirmed the positive relationship between people’s PET and pro-environmental behaviors [68,69]. People who have experienced environmental pollution are more willing to take action to solve environmental problems due to their increased perception of environmental threats [70,71,72]. In terms of green consumption behavior, when consumers personally perceive environmental pollution problems, they show a higher willingness to purchase green products and participate in recycling [73]. The research of Soares et al. [74] shows that when consumers perceive the environmental pollution risk of plastic products, they are more willing to purchase fewer plastic products or buy degradable substitutes. Schmitt et al. [69] found that people with higher PET are more likely to perform behaviors such as purchasing organic food or energy-efficient appliances. Therefore, the following hypotheses are proposed

**Hypothesis** **7 (H7).** *Higher PET can directly improve green consumption behavior.*

**Hypothesis** **8 (H8).** *Higher PET can improve green consumption behavior by improving the intention.*

**Hypothesis** **9 (H9).** *Internet use impacts green consumption behavior through the mediation of PET.*

Meanwhile, an individual’s PET is related to the nature of environmental events [75]. Compared with social and environmental events, consumers pay more attention to environmental issues that are related to their own interests [76,77]. When individuals believe that a threat is not personal but a broader social problem, individuals will expect the public to share the responsibility and thus have little incentive to take personal actions to reduce the risk [78], so they lack the corresponding motivation to take action. This may result from different environmental values of anthropocentric values and ecocentric values [79]. Due to anthropocentric values, those environmental issues that are more related to individuals’ health, life, and well-being will stimulate higher PET and then affect an individual’s willingness to engage in pro-environmental behaviors [75,80,81]. However, multiple studies also proved the important role of environmental values and ecocentric values in promoting green behaviors [82,83], indicating that both anthropocentric values and ecocentric values can promote an individual’s green behaviors. Therefore, this study divides PET into personal PET and public PET to explore the impacts of different perceived threats on green consumption behavior. Personal PET refers to the perceived threats of environmental problems to consumers’ own health and living environment (air pollution, garbage pollution, etc.), while public PET refers to the perceived threats of environmental problems that are less relevant to consumers’ own living environment (climate change, destruction of forests, etc.).

**Figure 1:**
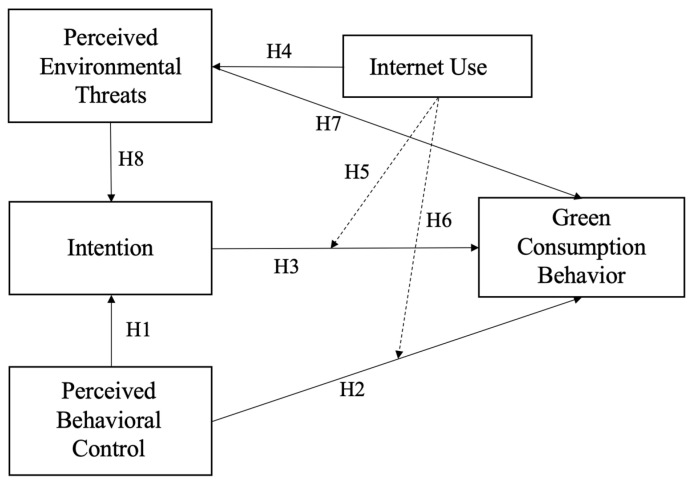
Proposed Framework (Note: Solid lines refer to casual relations, dotted lines refer to moderation effects).

Based on the hypothesis, the theoretical framework of this study is proposed, as shown in Figure 1.

## 3. Materials and Methods

### 3.1. Measures and Data Collection

In this study, data were collected through an online questionnaire. The questionnaire consists of two parts. The first part collected demographic information of respondents, including age, gender, education, and geographic area. The second part included questions related to the variables of the study. Measures of the variables are in the form of a five-point Likert scale, with 1 = Strongly Disagree to 5 = Strongly Agree. Green consumption intention and PBC consist of three and four items, respectively, adopted from Paul et al. [84] and Yadav and Pathak [49]. Measures of green consumption behavior consist of four items adopted from Gong [34] and Liobikienė et al. [85], asking respondents about the frequency of their green consumption behaviors (1 = Never to 5 = Very Frequently). Measures on Internet use asked respondents about their Internet use frequency in the past year (1 = Never to 5 = Very Frequently) [37,71]. Measures on PET asked respondents about the severity of environmental problems they perceive [31] (1 = No Such Problem to 5 = Extremely Serious). Among the items of PET, air pollution, water pollution, noise pollution, industrial waste pollution, household waste pollution, and green space shortage are classified as personal PET in data analysis. Forest destruction and climate change are classified as public PET in data analysis. The questionnaire was tested in a pilot survey in July 2022 with 56 respondents. Most of them are college students in Beijing. The measures were then adjusted and tuned based on the results of the pilot study and expert opinions (shown in Appendix A).

The aim of the study is to investigate the green consumption intention–behavior gap of general Chinese consumers. To obtain more representative samples, stratified random sampling was adopted, dividing Chinese consumers into those from the western, middle, and eastern parts of China. Respondents were chosen randomly from the sample pool of the Wenjuanxing platform, a mainstream questionnaire website in China, which enables pushing questionnaires to respondents in the three areas, covering a wide range of samples [14,86]. According to Hair et al. [87], the minimum sample size of PLS-SEM analysis is ten times larger than (1) the largest number of measures of a construct or (2) the largest number of direct paths of a latent construct. Therefore, this study requires a minimum sample size of 80 (8 × 10). However, to improve sample representativeness and avoid bias due to a small sample size, a total of 507 questionnaires were distributed from 28 July 2022 to 10 August 2022. Respondents had a chance to win rewards, such as shopping coupons, discount cards, or a small cash voucher after finishing the questionnaire.

A total of 419 valid responses were collected after removing those which failed to pass the attention screening. Detailed demographic information is presented in Table 2. Among the 419 samples, males account for 48.96% (n = 204), and females account for 51.31% (n = 215). Gender distribution is relatively balanced. In terms of age, people aged 21–35 account for 71.60%, people aged 36–50 account for 19.33%, and young and middle-aged people account for the majority of the respondents. About 60% of interviewees are from the eastern part of China, which is close to the trend of the actual population distribution of the country.

### 3.2. Data Analysis

PLS-SEM is a data analysis method suitable for exploratory studies with a focus on prediction and theory development [87,88]. This study extended the TPB model by adding new variables into the framework; therefore, given the theoretical exploration nature of the study, the PLS-SEM method was used for data analysis. Moreover, Internet use is a single-item construct, which is acceptable in PLS-SEM analysis but not suitable for the CB-SEM method, which has a restriction on the number of items of a construct [87]. Thus, the PLS-SEM method is used in this study.

First, SmartPLS software was used to evaluate the reliability and validity of the measurements of the research model. Second, the path coefficients of the structure model and the significance of the paths were analyzed, and the hypotheses were verified. Third, based on the structure model results, the consumer’s decision-making process on green consumption behavior in the TPB model was explained, including the moderating effects of Internet use on the relationship between PBC and behavior as well as the relationship between intention and behavior. Finally, the mediating effect of PET on the relationship between Internet use and green consumption behavior was verified with the Bootstrapping method using Stata software.

## 4. Results and Discussion

### 4.1. Model Reliability and Validity

Table 3 shows the reliability and validity of each construct. First, factor loadings of most items are higher than, or very close to, the recommended level of 0.70, indicating acceptable internal reliability. Second, Cronbach’s α of each latent variable is higher than or close to the standard of 0.8, and the composite reliability (CR) is higher than the acceptable value of 0.70, indicating good consistency of each construct [89]. The AVE values range from 0.52 to 0.76, exceeding the threshold of 0.50 [90]. Therefore, each construct in this study has good convergent validity.

Discriminant validity is assessed through the Heterotrait-monotrait (HTMT) ratio, as shown in Table 4. The HTMT value of each construct is smaller than the threshold of 0.85, indicating that the constructs of latent variables in this study have acceptable discriminant validity [91].

In terms of model fit, R^2^, Q^2^, and f^2^ are tested. Following the criteria of Chin and Marcoulides [92], an R^2^ of 0.333 indicates moderate explanatory power. In previous consumer studies that used an extended TPB model to study green consumption behaviors, R^2^ values ranging from 0.30 to 0.40 were considered to have good explaining power [12,13,43]. In this study, the R^2^ value is 0.340 for behavior and 0.493 for intention, explaining more than 34.0% of green consumption behavior and more than 49.3% of green consumption intention. Therefore, the proposed model of the study has good explaining power.

Q^2^ is an indicator of the predictive relevance of a model with the threshold value of Q^2^ > 0 [88]. Based on the Blindfolding analysis, the Q^2^ values of constructs in this study are 0.183 for behavior and 0.373 for intention, indicating that the model has good predictive relevance.

The f^2^ values of BH’s independent variables are 0.107 for intention, 0.036 for PBC, and 0.056 for PET. According to the criteria of Chin and Marcoulides [92], f^2^ values between 0.020 and 0.150 indicate that these three predictor variables have relatively small but acceptable effects in the structural model to predict behavior.

This study used the variance inflation factor (VIF) to check common method bias issues. According to Hair et al. [87], the VIF values should be less than 5.00. The VIF value of each item in our study is under this threshold, as shown in Table 3, indicating that common method bias is not a concern in this study.

### 4.2. Structural Results

**Figure 2:**
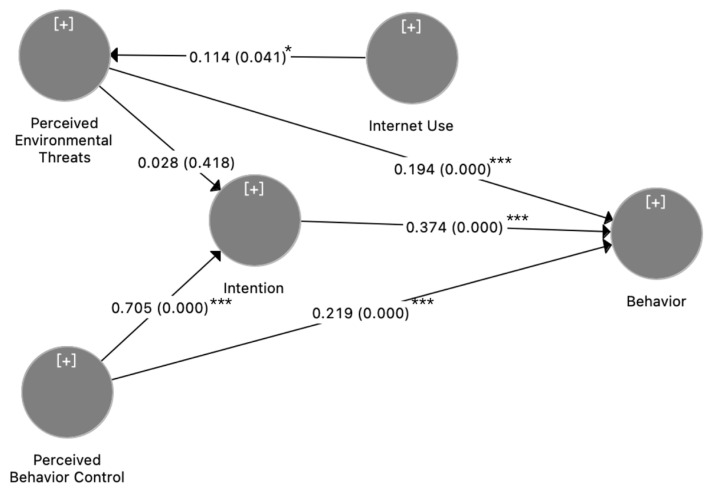
Research Model and Structural Results (Note: *** *p* < 0.001, * *p* < 0.05).

Structural results of the theoretical model are presented in Figure 2 and Table 5. The data were analyzed in three models based on the different PET mentioned above. Model 1 examined the structure model with total PET, Model 2 examined with personal PET, and Model 3 examined with public PET. Results of Model 1 show that PBC positively influences consumers’ intention (β = 0.705, *p* < 0.001). Intention (β = 0.374, *p* < 0.001) together with PBC (β = 0.219, *p* < 0.01) can directly influence behavior. Thus, H1–H3 are supported, indicating that the theory of planned behavior has a good ability to explain an individual’s green consumption behavior. Meanwhile, Internet use can positively improve total PET (β = 0.114, *p* < 0.05) and personal PET (β = 0.112, *p* < 0.05) but shows no significant effect on public PET (*p* > 0.05). Thus, H4 is partially supported.

Structural results of all three models show that PET can significantly and directly promote green consumption behavior (*p* < 0.001); therefore, H7 is supported. However, neither personal PET nor public PET has a significant positive effect on intention, suggesting that consumer PET does not affect green consumption behavior through the mediating effect of intention. Therefore, H8 is not supported. A possible reason is that the threat of environmental pollution arouses an individual’s negative emotions, such as guilt and fear, urging individuals to make corresponding actions, such as sacrificing part of their own interests to purchase more environmentally friendly choices (such as higher prices of green products than its ordinary substitutes) [93]. In contrast, intention emphasizes the motivation of an individual’s intention, which is largely influenced by a positive attitude. The negative emotional factors evoked by PET may be different from the positive emotional factors; thus, PET acts directly on behavior rather than through intention. This direct impact result is in line with the study of Schmitt et al. [69].

### 4.3. The Moderating Effect of Internet Use

**Figure 3:**
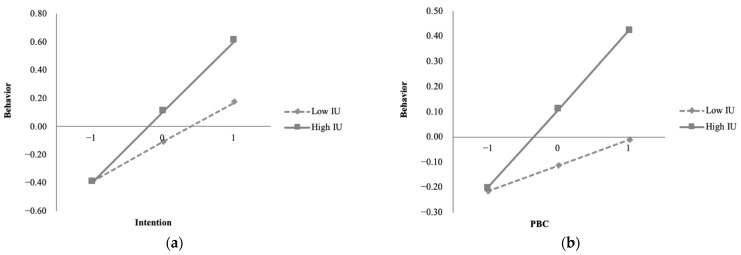
Moderation Analysis (**a**) Moderation effect of Internet use on the relationship between intention and behavior (**b**) Moderation effect of Internet use on the relationship between PBC and behavior.

The Moderation Effect function in SmartPLS software was used to test the moderating effect of Internet use on green consumption behavior. As shown in Table 6 and Figure 3, Internet use has significant moderating effects on the path of PBC–green consumption behavior (*p* < 0.05) and the path of intention–green consumption behavior (*p* < 0.01). The moderating effects are significantly positive, with the slopes of both pathways increased. At a higher level of Internet use, PBC, as well as intention, has stronger positive impacts on behavior; thus, H5 and H6 are supported. Meanwhile, the moderation effect of Internet use on the relationship between PBC and behavior (β = 0.110) is stronger than that between intention and behavior (β = 0.105), given that the path coefficient of the former is larger. The results indicate that the use of the Internet strengthens the influence of PBC on green consumption behavior and intention on behavior, which promotes the conversion of PBC to green consumption behavior, and intention to green consumption behavior. Therefore, Internet use is conducive to closing the intention–behavior gap.

### 4.4. The Mediating Effect of PET

Structural results of Model 2 and Model 3 in Table 5 indicate that both personal PET (β = 0.164, *p* < 0.001) and public PET (β = 0.176, *p* < 0.001) can improve consumers’ green consumption behavior. However, Internet use can affect green consumption behavior by improving personal PET (β = 0.112, *p* < 0.05) but has no significant effect on public PET (*p* > 0.05). To test the mediation effect of personal PET on the relationship between Internet use and behavior, a Bootstrapping method is employed, using path analytic procedures to test the significance of the PET mediator. The Bootstrapping method is considered a more reliable technique for testing mediation effects without the need for discrete hypothesis tests about components of the model [94,95]. This method has been applied in recent studies on consumer’s sustainable behaviors [96,97].

The results of Bootstrapping in Table 7 further verify the mediating effect of personal PET on the relationship between Internet use and green consumption behavior. Results show that the mediating effect of personal PET is significant with a 95% CI (confidence interval) of (0.003, 0.059); that is, Internet use could promote green consumption behavior by improving consumers’ personal PET. On the contrary, overall PET and public PET did not play a mediating role in this relationship, with 95% CI of (−0.003, 0.044) and 95% CI of (−0.004, 0.026), respectively. Therefore, Internet use enhances green consumption behavior through the mediator of consumers’ personal PET, H9 is partially supported.

### 4.5. Summary of the Hypotheses Test Results

As shown in Table 8, research findings based on the data analysis and results of the hypotheses test are reported.

## 5. Discussion and Implications

### 5.1. Discussion

#### 5.1.1. Structural Results

The study proposed a theoretical framework integrating the TPB model with the variables of Internet use and PET to investigate the impact of Internet use on closing the intention–behavior gap. The results of H1–H3 show that intention and PBC are good predictors of green consumption behaviors, which is consistent with previous studies of extended TPB [13,43]. The results of H7 show that consumers will take corresponding actions of purchasing in a green manner due to threats that arose from the degradation of the environment. H8 further proves that PET impacts behavior directly rather than through intention, which is in line with and further complement the study of Schimitt et al. [69]. With the development of Internet media, more intensive Internet use also impacts how consumers perceive environmental problems, especially those related to their own interests, as the result of H4 indicated. This finding is consistent with the study of Liu et al. [31]. Furthermore, the model still has good model reliability and validity after integrating new variables of Internet use and PET, indicating the proposed framework’s good theoretical explaining power.

#### 5.1.2. Moderating Effect of Internet Use

As the result of H5 shows, in the process of intention transforming to behavior, high frequency of Internet use can make consumers more conscious of the severity of environmental problems, realizing that human interests are closely related to the environment. Such information can enhance consumers’ environmental awareness and awaken their intention of environmental protection, which promotes more practice of green consumption behaviors such as purchasing more green products and recycling packages.

As the result of H6 shows, in the process of PBC transforming to behavior, the use of the Internet can improve the necessary ability of consumers to implement green behaviors. By gaining more environmental knowledge through the Internet, consumers can identify what is a green product, identify the green label, and understand the consequences of their behavior on the environment. By improving the ability of consumers to conduct green consumption, Internet use can promote consumers’ green consumption behavior.

This result of moderation analysis shows that Internet use is an important external information factor in consumers’ green consumption decision-making process; this result is in line with the study of Bedard and Tolmie [30] and the study of Xiao et al. [33]. The finding further extends and complements previous research that Internet use can significantly close the intention–behavior gap. Internet media has created an information-rich environment in which sufficient information and knowledge from this channel has subtly changed the knowledge structure of consumers. Only when consumers understand the relevant concepts of green consumption and their behavioral consequences are they more likely to identify, evaluate, and finally, purchase green products. Therefore, the acceptance of H5 and H6 provides a theoretically possible solution that the rational use of the Internet can help reduce the green consumption intention–behavior gap and promote the sustainable transformation of individual’s behavior.

#### 5.1.3. Mediating Effect of PET

The results of H7 show that both personal PET and public PET can improve consumers’ green consumption behavior. In general, when people perceive a risk to be detrimental to their own interests, they are more willing to take action accordingly to prevent their interests from being harmed. Therefore, when consumers’ personal PET is enhanced by external information intervention, environmental consequences of their purchasing behaviors will become important considerations in the purchasing behavior. As for public environmental problems such as global warming and ecological diversity loss, individuals may take corresponding countermeasures out of environmental awareness and altruism [82]. This result is in line with previous studies [68,76].

However, when it comes to using Internet media to obtain information, results of H9 show that Internet use only affects green consumption by improving personal PET (β = 0.112, *p* < 0.05) but has no significant effect on public PET (*p* > 0.05), which indicates that consumers pay more attention to environmental issues online that are closely related to their own living conditions, such as local air pollution and household waste pollution. Given the community characteristic of social networks, information exchange through the Internet is to communicate with or attract the attention of those people with shared interests. Therefore, Internet media tend to report local environmental news more related to individual health and welfare, which is more likely to attract readers’ attention, and readers are more likely to be attracted by this news and have better responses. In contrast, environmental knowledge and environmental issues with more public attributes, such as carbon emissions and global warming, are typically acquired through traditional school education or traditional media, such as television and news reports, rather than through Internet channels with more obvious attributes of “we media” [98,99].

### 5.2. Implications

This study can contribute to the current research in several ways. First, in terms of theoretical implications, the study proposes a framework based on TPB to predict green consumption behavior. Specifically, Internet use is covered as a new contextual variable to moderate the relationship between intention and behavior as well as that between PBC and behavior. Meanwhile, PET is integrated as a mediator to explain how Internet use impacts behavior. The study complements the current TPB research by addressing the intention–behavior gap issue and contributes to the current literature by integrating Internet use and PET.

Second, the study also offers some managerial implications for managers and decision-makers. The results of this study show that Internet media can promote consumers’ green consumption behavior and close the intention–behavior gap. Therefore, when promoting green products, the positive role of the Internet in spreading information and educating consumers should be fully explored. Managers and decision-makers could consider using Internet channels to commercialize and promote green products to reach target consumers better. The results of H6 indicate that Internet use can increase the dissemination of environmental knowledge, which is beneficial to impacting the consumer’s decision-making process by influencing the consumer’s PBC, that is, the consumer’s perceived difficulty in purchasing and perceived control over the behavioral consequences. Therefore, relative product information making consumers feel fewer purchase barriers and better purchase consequences can be conveyed to consumers through the online channel, such as the concept of green products, the identification of green labels, and the recycling of product packaging, as well as the positive consequences of purchasing green products for both personal and social benefits.

Third, the study also provides some implications for policymakers in that Internet media needs to be guided in the way of reporting environmental issues. Results of this research show that Internet media reporting on environmental issues can promote individuals’ green behavior by increasing their PET. This conclusion is mainly applicable to environmental issues that are closely related to individuals’ health, well-being, and personal interests. Therefore, policymakers should encourage Internet media to continue promoting individual behavior change by disseminating environmental issues relevant to personal benefits and leveraging people’s willingness to protect their own welfare and interests. Furthermore, the result of H9 suggests that the Internet plays little role in promoting green purchasing behavior change through arousing public PET. Due to the nature of the Internet of chasing hotspots, when spreading public environmental issues that have little to do with individuals, the attention it receives from readers is not as much as that from spreading personal environmental issues such as local pollution outbreaks. However, public PET can also significantly drive consumer behavior change; therefore, policymakers should attach great importance to the role of public PET, regulate and urge Internet media to take social responsibility, and report both public and personal environmental issues rather than personal ones only to seek high exposure. Moreover, some consumers may believe in their important role in contributing to solving environmental issues, but a lack of clear and detailed instructions on what they can do and how may hinder their willingness to act on actual consumption behaviors. Therefore, when educating consumers, policymakers should consider not only stressing the importance of green consumption but also informing consumers with clear and practical advice on how to perform green consumption through the channel of Internet media, which enables the dissemination of information in a faster and easier to understand manner.

### 5.3. Limitations and Future Research

There are some deficiencies of this study to be solved. First, the data were collected in China and considered general Chinese consumers. Thus, future research could consider the diverse development level of the Internet infrastructure, designing a comparative study investigating geographic heterogeneity across the country of Internet use’s impact on consumer’s green consumption behaviors, which may provide more practical and precise managerial implications in different areas. Moreover, the generality and to what extent the results of this study are applicable in other contexts await to be further proven. Therefore, future research could also consider a comparative study investigating this framework in different Asian countries and test the framework in different cultural and economic contexts.

Second, due to the constraints of resources and time, this study collected data through online questionnaires and used cross-sectional data for analysis. The self-administrated questionnaire method, although it is efficient in collecting data, may lead to self-report bias. Therefore, future research could first consider utilizing actual consumption data to capture the precise behaviors of consumers better. Moreover, future research could also conduct a longitudinal study to repeat the survey several times and track the respondents’ responses over time, which is conducive to investigating the changes of such moderation and mediation effects with the increasing penetration of the Internet. Furthermore, future research could enlarge the sample size to enhance the representativeness of the data.

Third, this study used PET as a mediator and Internet use as a moderator to explain how the Internet can help close the intention–behavior gap. Due to the complex nature of consumers’ decision-making processes, more psychological, as well as contextual factors other than PET and Internet use, could also have significant impacts on the intention–behavior gap. Future studies could consider adding other factors into the framework, such as consumers’ environmental knowledge obtained during Internet use and the changes in perceived effectiveness in contributing to sustainable development, to improve the model’s predicting power and to better address the intention–behavior gap.

## 6. Conclusions

This study proposed a framework based on the theory of planned behavior to study the theoretical mechanism of Internet use on closing the green consumption intention–behavior gap from the perspective of information media. The results of PLS-SEM analysis show that Internet use can promote individual green consumption behavior and significantly reduce the intention–behavior gap through two pathways, including the moderation effect of Internet use and the mediation effect of PET.

First, through the moderating effect, Internet use can significantly promote the conversion of intention to green consumption behavior (*p* < 0.01) and the conversion of PBC to green consumption behavior (*p* < 0.05) so as to reduce the intention–behavior gap. Second, through the mediating effect, Internet use can also promote green consumption behavior through the mediator of perceived environmental threats, especially personal perceived environmental threats.

The results show the important role of Internet media in contributing to the promotion of green consumption; therefore, the Internet as an information medium should be reasonably utilized to increase the dissemination of relevant environmental knowledge. At the same time, it is necessary to guide the way Internet media reports environmental news. In addition to arousing people’s awareness through reporting on the environmental pollution crisis, it is also necessary for the Internet media to provide practical information on how to perform green consumption behavior to guide the green transformation of an individual’s consumption patterns.

## Figures and Tables

**Table 1:** Green Intention, Behaviors, and Intention–Behavior Gap.

Country	Guiding Theory	Sample	Method	Dependent Variable	Major Significant Moderating/Mediating Constructs	Sources
China	TPB	572	SPSS/ SEM	Recycling Behavior	Proactive Personality, Price Sensitivity	[14]
China	TPB	156	SEM	Green Regeneration of Industrial Brownfields	Awareness of Consequences, Ascription of Responsibility	[12]
Australia	TPB	772	SPSS	Pro-Environment Behaviors	Actual Behavior Control, Implement Intention, Situational Context	[39]
U.S.	None	307	Regression	Green Hotel Booking	Comparable Price, Green Certification	[15]
Vietnam	None	431	SEM	Green Consumption Intention	Social Media, Eco-Brand, Environmental Concern, Eco-Label	[32]
Singapore	TPB	1168	Regression	Green Buying and Civic Engagement	Media Dependency, Traditional media attention, Interpersonal Communication	[40]
Australia	TPB	1011	PLS-SEM	Organic Food Consumption	Communication, Trust, Satisfaction	[17]
Bangladesh	TPB/The Protection Motivation Theory	305	PLS-SEM	Green Buying Behavior	Attitude	[13]
China	TPB	243	SEM/Regression	Pro-Environmental Road Freight Transportation Behaviors	Perceived Policy Effectiveness	[41]
Thailand	TPB	447	Regression	Green Purchasing Behavior	Green Value, Environmental Knowledge	[38]
Germany	None	99	Regression	Organic Grocery Purchasing	Perceived Informational Purchase Barriers, Product Category Involvement, Health Consciousness	[29]
Bangladesh	TPB	365	SEM	Buying Intention of Energy-Efficient Home Appliances	Green Self-Identity	[11]

**Table 2:** Demographic Information of Interviewees.

Variable	Category	Number	Percentage (%)
Gender	Male	204	48.69%
	Female	215	51.31%
Age	20 and Under	10	2.39%
	21–35	300	71.60%
	36–50	81	19.33%
	51–65	25	5.97%
	65 and Above	3	0.72%
Geographic	East Area	267	63.72%
	Middle Area	71	16.95%
	West Area	81	19.33%
Average Monthly Household Income	1000 RMB and Under	3	0.72%
	RMB 1000–3000	32	7.64%
	RMB 3000–5000	64	15.27%
	RMB 5000–8000	123	29.36%
	RMB 8000–10,000	77	18.38%
	RMB 10,000 and above	120	28.64%
	Total	419	

**Table 3:** Reliability and Validity of Measurement Items.

Variable	Item	Factor Loadings	Cronbach’s Alpha	CR	AVE	VIF
Behavior	BH1	0.76	0.75	0.84	0.57	1.44
	BH2	0.84				1.80
	BH3	0.67				1.41
	BH4	0.74				1.55
Intention	INT1	0.89	0.84	0.91	0.76	2.22
	INT2	0.83				1.76
	INT3	0.90				2.30
Perceived Environmental Threats	PET1	0.74	0.87	0.90	0.52	1.76
	PET2	0.75				2.01
	PET3	0.73				1.62
	PET4	0.76				2.00
	PET5	0.78				1.92
	PET6	0.68				1.68
	PET7	0.65				1.60
	PET8	0.70				1.59
Perceived Behavioral Control	PBC1	0.79	0.83	0.89	0.66	1.59
	PBC2	0.83				1.84
	PBC3	0.85				2.32
	PBC4	0.79				1.95

Note: BH = behavior, INT = intention, IU = Internet Use, PET = perceived environmental threats, PBC = perceived behavioral control.

**Table 4:** Discriminant Validity-HTMT.

	Behavior	Intention	Internet Use	PBC	PET
Behavior					
Intention	0.639				
Internet Use	0.180	0.046			
PBC	0.587	0.833	0.058		
PET	0.211	0.075	0.117	0.100	

**Table 5:** Results of Structural Analysis.

Path	Model 1 (Total PET)			Model 2 (Personal PET)			Model 3 (Public PET)		
	Path Coefficients	Std. D	*p*-Value	Path Coefficients	Std. D	*p*-Value	Path Coefficients	Std. D	*p*-Value
INT -> BH	0.374	0.062	0.000 ***	0.374	0.062	0.000 ***	0.381	0.063	0.000 ***
IU -> PET	0.114	0.056	0.041 *	0.112	0.055	0.041 *	0.088	0.052	0.089
PBC -> BH	0.219	0.063	0.000 ***	0.207	0.063	0.001 **	0.216	0.063	0.001 **
PBC -> INT	0.705	0.034	0.000 ***	0.704	0.034	0.000 ***	0.704	0.034	0.000 ***
PET -> BH	0.194	0.042	0.000 ***	0.164	0.041	0.000 ***	0.176	0.043	0.000 ***
PET -> INT	0.028	0.034	0.418	0.033	0.033	0.330	0.006	0.038	0.865

Note: *** *p* < 0.001, ** *p* < 0.01, * *p* < 0.05.

**Table 6:** Moderation Analysis.

Moderator	Path	Coefficients	Std. D	*p*-Value
IU	PBC -> BH	0.105	0.045	0.019 *
	INT -> BH	0.110	0.038	0.004 **

(Note: ** *p* < 0.01, * *p* < 0.05).

**Table 7:** Mediation Analysis.

	Model 1 (Total PET)			Model 2 (Personal PET)			Model 3 (Public PET)		
	Path Coef.	Std. Error	95% CI	Path Coef.	Std. Error	95% CI	Path Coef.	Std. Error	95% CI
Indirect Effect	0.014	0.011	(−0.003, 0.044)	0.021	0.132	(0.003, 0.059)	0.006	0.007	(−0.004, 0.026)
Direct Effect	0.190	0.052	(0.087, 0.299)	0.184	0.056	(0.073, 0.302)	0.200	0.058	(0.081, 0.309)

**Table 8:** Hypotheses Test Results.

Hypothesis	Proposition	Result
H1	PBC positively impacts green consumption intention	Supported
H2	PBC positively impacts green consumption behavior	Supported
H3	Intention positively impacts green consumption behavior	Supported
H4	Internet use positively impacts PET	Partially Supported
H5	Internet use can positively moderate the relationship between intention and green consumption behavior	Supported
H6	Internet use can positively moderate the relationship between PBC and green consumption behavior	Supported
H7	Higher PET can directly improve green consumption behavior	Supported
H8	Higher PET can improve green consumption behavior by improving intention	Not Supported
H9	Internet use impacts green consumption behavior through the mediation of PET	Partially Supported

## Data Availability

The data that support the findings of this study are available from the corresponding author upon reasonable request.

## Appendix A

**Table A1:** Measurement Items.

Variable	Item	Sources
Behavior	BH1: How often do you purchase products with green labels	[34,85]
	BH2: How often do you purchase products with recyclable packaging (e.g., glass jars)	
	BH3: How often do you bring your own baskets or bags when shopping for daily necessities	
	BH4: How often do you recycle the plastic bags	
Internet Use	IU: How often do you use the Internet, including through mobile phone, for the past year	[31]
Intention	INT1: I am willing to purchase green products	[49,84]
	INT2: I plan to purchase green products	
	INT3: I will make an effort to purchase green products	
Perceived Environmental Threats	PET1: How’s the severity of air pollution in your area	[31]
	PET2: How’s the severity of water pollution in your area	
	PET3: How’s the severity of noise pollution in your area	
	PET4: How’s the severity of industrial waste pollution in your area	
	PET5: How’s the severity of domestic waste pollution in your area	
	PET6: How’s the severity of green space shortage in your area	
	PET7: How’s the severity of forest destruction in your area	
	PET8: How’s the severity of climate change in your area	
Perceived Behavioral Control	PBC1: I have resources, knowledge and abilities to purchase green products	[49,84]
	PBC2: If it were entirely up to me, I am confident that I will purchase green products instead of conventional products	
	PBC3: I am capable of purchasing green products	
	PBC4: There are likely to be plenty of opportunities for me to purchase green products	
